# C3a and C5a plasma concentrations in patients with diabetic retinopathy: a cross-sectional study

**DOI:** 10.1186/s40942-026-00821-6

**Published:** 2026-03-13

**Authors:** Paula Basso Dias, Iara Messias-Reason, Germano Ramos Boff, Kenzo Hokazono, Renato Nisihara

**Affiliations:** 1 Internal Medicine Post Graduate, Clinical Hospital, Federal University of Paraná, Curitiba, Brazil; 2 Department of Ophthalmology, Clinical Hospital, Federal University of Paraná, Curitiba, Brazil; 3 Department of Medicine, Positivo University, Curitiba, Brazil; 4 Mackenzie Evangelical School of Medicine, R. Padre Agostinho 2770, Curitiba, 80730-000 Brazil

**Keywords:** Complement system, Diabetic retinopathy, Anaphylatoxins, C3a, C5a

## Abstract

**Background:**

Diabetic retinopathy (DR) is the leading cause of irreversible blindness in adults, with inflammation playing a critical role in its pathogenesis, particularly involving the complement system (CS). Components of the complement cascade, such as C3a and C5a, have been identified in both vitreous and plasma samples from individuals with DR. This study aimed to evaluate plasma concentrations of C3a and C5a in relation to DR onset and severity.

**Methods:**

A total of 150 patients with diabetes mellitus (DM) underwent ophthalmic examination and were categorized into groups: without DR (control group), mild/moderate/severe non-proliferative DR (NPDR) and proliferative DR (PDR). Plasma C3a and C5a levels were measured using sandwich enzyme-linked immunosorbent assays (ELISA).

**Results:**

Significant elevations in C3a levels were found in severe NPDR (*p* = 0.02) and PDR (*p* < 0.05) groups compared to controls. Furthermore, severe/proliferative DR exhibited higher C3a levels than mild/moderate NPDR (*p* = 0.015). No significant differences in C5a concentrations were observed among the groups.

**Conclusion:**

These findings suggest the involvement of complement in the pathogenesis of DR, and the association of C3a with DR severity highlights its potential as a prognostic biomarker and therapeutic target.

## Background

Diabetic retinopathy (DR) affects more than 60% of individuals with type 2 diabetes and is the leading cause of irreversible blindness in adults [1]. DR can be classified into mild, moderate, and severe non-proliferative diabetic retinopathy (NPDR), and proliferative diabetic retinopathy (PDR), which carries a heightened risk of vision loss [2, 3]. While factors such as vascular regeneration dysfunction, oxidative stress, and hyperosmolarity contribute to DR, inflammation involving the activation of complement system (CS) plays a crucial role in disease development [4–7].

The CS comprises over 40 plasma and membrane bound proteins that serves as a central mediator of inflammatory processes and innate defense mechanisms. The activation of complement occurs through the alternative, classical, and lectin pathways, which leads to the generation of distinct effectors, including the anaphylatoxins C3a and C5a that have important proinflammatory role. Activation of complement can also generate opsonins (C3b, iC3b, and C3d), which mediate the removal of distinct targets and immune complexes; and the terminal membrane attack complex (MAC, C5b-9) that can disrupt cell membranes causing lysis [5].

CS components can be locally synthesized by microglia, macroglia, and retinal pigment epithelium (RPE), suggesting an additional role in retinal protection [8]. Furthermore, the CS is implicated in various diabetic complications, including nephropathy, and has been linked to ocular disorders such as age-related macular degeneration (AMD) and uveitis [9, 10]. In patients with DR, the retinal tissue frequently exhibits ischemic conditions that promote CS activation [11].

The activation fragments, C3a and C5a, can promote inflammation and activate cells expressing G protein-coupled anaphylatoxin receptors, C3aR and C5aR [12]. Activation of C3a in endothelial cells initiates a cascade amplifying complement activation, implicated in microvascular thrombosis [13]. C5a, a potent chemotactic molecule, induces vascular endothelial growth factor (VEGF) expression and promotes angiogenesis in the retina [14]. Both C3a and C5a have been detected in the vitreous and plasma of patients with DR [15–20]. However, there are no studies analyzing serum concentrations of these molecules in DR patients classified according to disease severity. Such information may be valuable in establishing C3a and C5a as possible non-invasive biomarkers and therapeutic targets for DR.

The objective of this study was to determine whether plasma concentrations of C3a and C5a influence the onset or severity of DR.

## Methods

### Ethical issues

This is a cross-sectional study, approved by the Institutional Research Ethics Committee under protocol n^o^ 329.141. All participants signed an informed consent. The study was conducted according to the Good Clinical Practice guidelines and the Declaration of Helsinki.

### Sample

A convenience sample comprising 150 patients diagnosed with diabetes mellitus (DM) and receiving regular ophthalmological care at the Hospital de Clínicas - Universidade Federal do Paraná over a one-year period (October/2022-October/2023), as recruited sequentially based on their appointment schedule to participate in this study. Patients underwent a medical history assessment that included inquiries about sex, age, duration of disease, age at diabetes mellitus (DM) diagnosis, type of DM, presence of systemic arterial hypertension (SAH), and diabetic nephropathy. Additionally, the latest values of glycated hemoglobin and fasting blood glucose levels were verified for each patient.

The study included patients aged over 18 years diagnosed with either type 1 or type 2 diabetes mellitus according to the American Diabetes Association criteria. Exclusion criteria included individuals with media opacities that hindered adequate visualization of the fundus and precise classification of DR stages, as well as patients with other retinal diseases (e.g., AMD, uveitis), recent history of local or systemic inflammatory processes (< 3 months), and other chronic inflammatory conditions.

All participants underwent a comprehensive ophthalmic examination, which included evaluation of best-corrected visual acuity (BCVA), tonometry, slit lamp and fundus examination using a 78-diopter lens and indirect ophthalmoscopy after pupil dilation. DR was diagnosed based on the presence of characteristic lesions as defined by the *Early Treatment Diabetic Retinopathy Study* [2], including microaneurysms, hemorrhages, cotton-wool spots, intraretinal microvascular abnormalities, hard exudates, venous beading, and neovascularization. The severity of DR was classified as follows: no DR, mild NPDR, moderate NPDR, severe NPDR, and PDR.

### Plasma sample and ELISA assay

Venous blood samples were collected from the patients’ forearm on the day of ophthalmic evaluation. Following collection, the samples were centrifuged at 2500 g at 4 °C for 10 min, and the plasma was then separated and stored at -80 °C until analysis.

The circulating concentrations of C3a and C5a were determined using commercial “sandwich” enzyme-linked immunosorbent assays (ELISA). For C3a, the Human C3a ELISA Kit (ELK Biotechnology, Denver, CO) was employed, and for C5a, the BD OptEIA Human C5a ELISA Kit II (BD Biosciences, San Diego, CA) was used, following the manufacturers’ instructions. Plasma samples were diluted 1:20. Values were calculated using controls and calibration curves with standards provided by the kits. Inter- and intra-assay controls were employed in all assays.

### Statistical analysis

The statistical analysis was performed using Graphpad Prism (Graphpad Prism for Windows 5.03). Normality of distribution was assessed using the Shapiro-Wilk test. For the analysis of C3a and C5a values, the Student’s t-test was used for parametric independent variables, while the Mann-Whitney U test was employed for non-parametric independent variables. The correlation between C3a, C5a, and other non-parametric variables was evaluated using the Spearman’s rank correlation coefficient. A significance level of *p* < 0.05 was considered statistically significant.

## Results

### Studied sample

Among the 150 patients included in the analysis, 80 were female (53.3%). The mean age of the entire study population was 62.9 ± 10.18 years (range 18–80 years). Patients were categorized into five groups, with 25 patients classified as without DR (control group (CG). According to the severity of DR, patients were distributed as follows: 23 in the mild NPDR group, 23 in the moderate NPDR group, 25 in the severe NPDR group, and 54 in the PDR group. The average time since the diagnosis of DM was 16.4 years, with a mean age at DM diagnosis of 47 years. Table 1 shows the demographic characteristics, visual acuity data, and DR stages for each group.

**Table 1:** Clinical characteristics of the studied groups

	Without DR	Mild DR	Moderate DR	Severe DR	Proliferative DR	Total
Number of patients	25	23	23	25	54	150
Mean age (SD)	63.8 (11.4)	68.1 (10.7)	64.8 (7.2)	60.8 (10.0)	60.6 (9.8)	62.9 (10.2)
Diabetes Mellitus type 2 (%)	96%	95.6%	95.4%	91.3%	83.3%	91.3%
Number of women (%)	64%	56.5%	52.2%	52%	48.2%	53.3%
Mean HbA1c (SD)	7.7 (1.5)	7.6 (2.1)	8.1 (2.0)	8.2 (1.3)	8.6 (2.1)	8.2 (1.9)
Mean glycemia (SD)	150 (51.4)	135 (55.0)	155 (61.0)	167 (57.3)	168 (78.6)	156.9 (64.6)
Median of years of diagnosis	7.6	14.7	19.2	17.0	20.3	16.4
Median of age of diagnosis (years)	56.6	53.0	47.6	44.1	39.9	47.0
Mean visual acuity RE (SD)	0.9 (0.1)	0.6 (0.2)	0.6 (0.4)	0.4 (0.3)	0.2 (0.2)	0.5 (0.4)
Mean visual acuity LE (SD)	0.9 (0.1)	0.7 (0.2)	0.6 (0.3)	0.4 (0.3)	0.3 (0.3)	0.5 (0.3)
Hypertension (%)	72%	82.6%	81.8%	76%	87%	80.7%
Nephropathy (%)	8%	21.7%	19%	36%	40%	27.5%

DR= Diabetic retinopathy; SD= Standard deviation

### Data about C3a plasmatic concentration

We observed an association between higher levels of C3a and the presence of severe NPDR and PDR. As shown in Table 2, patients with severe NPDR had significantly higher mean C3a levels compared to those without DR (71.0 ± 24.0 ng/mL vs. 63.7 ± 11.1 ng/mL; *p* = 0.021). Similarly, patients with PDR also exhibited significantly elevated mean C3a levels compared to those without DR (69.8 ± 23.8 ng/mL vs. 63.7 ± 11.12 ng/mL; *p* = 0.04).

**Table 2:** Plasma Levels of C3a and C5a in the diabetic patients studied

	Without DR	Mild DR	Moderate DR	Severe DR	Proliferative DR	Without DR vs. Mild DR (*p* value)	Without DR vs. moderate DR (*p* value)	Without DR vs. severe DR (*p* value)	Without DR vs. Proliferative DR (*p* value)
Number of patients	25	23	23	25	54				
Mean C3a (SD)	63.7 (11.2)	57.9 (21.1)	67.0 (18.8)	71.0 (24.0)	69.8 (23.8)	*p* = 0.22	*p* = 0.43	*p* = 0.021	*p* = 0.043
Mean C5a (SD)	49.3 (13.0)	49.8 (17.6)	52.6 (18.1)	50.7 (16.2)	49.1 (16.1)	*p* = 0.71	*p* = 0.19	*p* = 0.46	*p* = 0.84

DR= Diabetic retinopathy; SD= Standard deviation

No significant differences in C3a levels were found between patients with mild NPDR and those without DR (57.9 ± 21.1 vs. 63.7 ± 11.12 ng/mL; *p* = 0.22), nor between patients with moderate NPDR and those without DR (67 ± 18.8 vs. 63.7 ± 11.12 ng/mL; *p* = 0.43), as observed in Table 2. Furthermore, analysis of disease severity revealed significantly higher C3a levels in patients with severe/proliferative disease compared to those with mild/moderate NPDR (70.1 ± 23.7 ng/mL vs. 62.5 ± 20.3 ng/mL; *p* = 0.015).

### Data about C5a plasmatic concentration

No significant differences were observed in C5a concentrations among patients with and without DR, with means of 49.8 ± 17.6 ng/mL in mild DR, 52.6 ± 18.1 ng/mL in moderate DR, 50.7 ± 16.2 ng/mL in severe DR, 49.1 ± 16.1 ng/mL in proliferative DR, and 49.3 ± 13 ng/mL in controls without DR (Table 2). C5a levels did not show any association with the development or severity of DR.

All variables were tested, and no significant correlations were found between plasma levels of C3a and C5a with sex, age, glycated hemoglobin, fasting glucose, and duration of disease in patients with DR.

## Discussion

There is an urgent need for innovative non-invasive strategies and the identification of novel therapeutic targets or prognostic biomarkers that could be used to effectively reduce the incidence and progression of DR in patients with DM. While evidence has demonstrated the role of complement components in the severity of DR [21], this study represents the first correlation in the literature between elevated C3a levels and RD severity.

The development of DM complications may be influenced by alterations in the production of proteins within the CS and its regulatory proteins [5, 22]. C3a and C5a, key anaphylatoxins of the CS, have been identified as elevated in patients with DR, one of the most devastating complications of DM [15–20]. Studies demonstrate the potential of the CS as a target for developing therapies that may aid in preventing diabetic vascular dysfunction [23, 24].

Established risk factors for proliferative DR include chronic hyperglycemia, longer diabetes duration, hypertension, and contributory environmental and genetic factors [25]. In our analysis, significantly higher levels of glycated hemoglobin and fasting glucose were observed in patients with more severe forms of DR compared to the CG. The study sample predominantly comprised patients with advanced DR (severe NPDR and PDR), typically more present in a tertiary care setting focused on managing patients requiring treatment.

This study demonstrated a significant elevation in plasmatic C3a levels among patients with severe NPDR and PDR compared to the CG. Previous research on vitreous humor from PDR patients identified local activation of complement components, with detection of fragments C3a, C5a, and Ba, along with consumption of C4, C5, and factor B [15]. Binding of C3a and C5a activates microglia and Müller cells, increasing production of IL-6 and VEGF, which enhance retinal endothelial cell permeability and proliferation, potentially damaging vascular endothelial cells and causing fluid leakage [5]. Additionally, fragments of the CS, such as C3d and C5b-9 complex, have been detected in the choriocapillaris of individuals with DR [16]. Smith et al. evaluated samples of aqueous and vitreous humor, and plasma from individuals with and without PDR, noting significantly higher levels of C3a in the aqueous and vitreous humor of PDR eyes [20].

In our study, no differences were observed in serum levels of C5a between patients with and without DR. Other authors indicate that the C5a fragment can influence inflammatory responses and angiogenic factor synthesis by binding to cellular receptors in the retina, triggering the release of IL-6 and VEGF [26]. Muramatsu et al. found elevated levels of C5a in the vitreous of PDR patients [17], while Manoharan et al. identified increased levels of factor D and C9 but did not find elevated C5a levels in the vitreous of PDR patients [18], a finding consistent with our study.

Zhang et al., compared the levels of plasmatic complement activation products C3a, C4a, and C5a of DR patients and controls [19]. Significant increases in C3a and C5a were observed in the RD group compared to controls, although severity of DR was not considered. Nevertheless, it is important to note that both this study and ours were limited to the analysis of plasma samples. It is important to consider that complement activation happens locally, with levels of C3a and C5a in the retina being substantially higher than those observed systemically, as demonstrated by others [15–18, 20].

The field of CS research is rapidly expanding in ophthalmology, encompassing diseases such as AMD, uveitis, and glaucoma [9, 27]. Recently, drugs like pegcetacoplan (SYFOVRE^®^) [28] and avacincaptad pegol (IZERVAY^®^) [29] have been approved by FDA bringing new treatment alternatives in the management of AMD geographic atrophy. These drugs target respectively, the C3 and C5 components, which are known to play critical roles in all pathways of CS activation. Given C3’s similar detrimental role in both DR and AMD pathogenesis, targeting C3 presents a promising avenue for DR treatment [23, 24]. Currently, approximately 40 clinical trials are investigating complement inhibitors across different ocular disorders [5].

This study has several limitations. Firstly, the limited sample size and the fact that it was conducted at a single center. Secondly, the study’s cross-sectional design limits our ability to establish causal relationships between variables. Thirdly, it is important to consider that patients with DM have various complications that can lead to inflammation and thus activation of the CS, including diabetic nephropathy and diabetic neuropathy [30, 31]. Moreover, it contributes to atheroma formation by secreting components of the alternative pathway by adipocytes and hepatocytes, thereby accelerating atherosclerosis [32]. Trying to diminish this bias, we included a control group of patients with DM but without DR due to the systemic inflammation characteristic of diabetic patients. Fourthly, we conducted serum analysis rather than vitreous analysis of complement activation. Vitreous analysis would allow us to assess local complement activation in the retina, but would not enable us to assess disease severity, as vitrectomy surgery is only performed in patients with advanced DR.

Despite solid evidence implicating the CS in DR, its clinical and therapeutic value remains incompletely established. Studies using the viral vector AAV2/8-sCD59 have shown promising reductions in vascular leakage, retinal ganglion cell apoptosis, and membrane attack complex (MAC) deposition in treated diabetic eyes compared to controls pre-injected with the virus [33]. Further studies are needed to elucidate the specific mechanisms of complement activation in the retina, aiming for a better understanding of its role in DR and to guide the development of targeted therapeutic agents.

## Conclusions

Significant increases in plasma levels of C3a have been observed in patients with severe non-proliferative diabetic retinopathy (NPDR) and proliferative diabetic retinopathy (PDR), suggesting involvement of complement activation in the pathogenesis of DR. No significant differences were observed in plasma concentrations of C5a between the study groups. The association of C3a with DR severity highlights its potential as a prognostic biomarker and potential therapeutic target.

## Data Availability

Data supporting this study’s findings are available from the corresponding author upon reasonable request and approval.

## Abbreviations

AMD: Age-related macular degeneration
BCVA: Best-corrected visual acuity
CG: Control group
CS: Complement system
DM: Diabetes mellitus
DR: Diabetic retinopathy
ELISA: Enzyme-linked immunosorbent assay
ETDRS: Early Treatment Diabetic Retinopathy Study
FDA: Food and Drug Administration
MAC: Membrane attack complex
NPDR: Non-proliferative diabetic retinopathy
PDR: Proliferative diabetic retinopathy
RPE: Retinal pigment epithelium
SAH: Systemic arterial hypertension
VA: Visual acuity
VEGF: Vascular endothelial growth factor
